# Teledermatology: From Prevention to Diagnosis of Nonmelanoma and Melanoma Skin Cancer

**DOI:** 10.1155/2011/125762

**Published:** 2011-07-11

**Authors:** G. Fabbrocini, V. De Vita, F. Pastore, V. D'Arco, C. Mazzella, M. C. Annunziata, S. Cacciapuoti, M. C. Mauriello, A. Monfrecola

**Affiliations:** Section of Dermatology, Department of Systematic Pathology, University of Naples Federico II, 80131 Naples, Italy

## Abstract

Telemedicine is a rapidly developing application of clinical medicine where medical information is transferred through interactive audiovisual media for the purpose of consulting remote medical procedures or examinations, reducing the time of consultation for patients. Teledermatology as an application of telemedicine was developed in 1995: it turns out to be a gradually more ordinary mean of delivering dermatologic healthcare worldwide and will almost certainly have a greater medical function in the future. In particular, teledermatology can aid in the prevention and diagnosis of nonmelanoma and melanoma skin cancer; telemedicine and teledermatology offer the opportunity to make available consultations with experts also by long distance. Overall, patients seem to accept teledermatology, considering it as an excellent mean to obtain healthcare, particularly in those areas with no expert dermatologists available. Clinicians have also generally reported affirmative experiences with teledermatology in the skin cancer field. Further studies focusing on cost effectiveness, patient outcomes, and patient and clinician satisfaction will facilitate to delineate the potential of teledermatology as a mean of prevention and diagnosis of nonmelanoma and melanoma skin cancer.

## 1. Introduction

Telemedicine is the practice of healthcare using interactive audio, visual, and data communication; this includes healthcare delivery, diagnosis, consultation, and treatment, as well as education and transfer of medical data [1]. Telemedicine may be as simple as two health professionals discussing a case over the telephone or as complex as using satellite technology and videoconferencing equipment to conduct a real-time consultation between medical specialists in two different countries. Telemedicine generally refers to the use of communications and information technologies for the delivery of clinical care. This medical practice has dramatically reduced the time of consultation for patients. 

Teledermatology as an application of telemedicine was developed in 1995 [2]: it turns out to be a gradually more ordinary mean of delivering dermatologic healthcare worldwide and will almost certainly have a greater medical function in the future. During the last years, teledermatology has gained a lot of interest among scientific community. It can deeply revolutionize the delivery of dermatology services, providing equitable services to remote areas and allowing primary care physicians to refer patients to well-equipped dermatological services at a distance [3]. Teledermatology uses telecommunication technologies to transfer information to patients via mobile phone and images of skin lesions to clinicians via the Internet. 

Teledermatology can also aid in prevention and diagnosis of skin cancer. Nowadays, a reduction of morbidity and mortality of nonmelanoma and melanoma skin cancer is the most important challenge for dermatology: teledermatology can be considered a new tool that allows preventing and diagnosing skin cancers.

## 2. Prevention

Because solar irradiance is the reason of many dermatologic effects, a certain number of studies have tried to evaluate different methods to calculate a daily parameter of sun irradiance. These studies have tried to correlate the daily parameter with human photoexposure in order to avoid sunburns and other sun risks [4–7]. Nevertheless, sunscreen use is often recommended in order to prevent certain types of skin cancers and sunburns [8, 9]. From a national Canadian questionnaire, it has been estimated that only a few part of population (about 20%) uses sunscreen regularly [10]. A major cause of nonadherence to sunscreen use is oversight, but also the misunderstanding about sunscreen uses and its benefit. In order to avoid the risks of sun exposure, many educational programs are in progress to inform people about preventive measures. Many scientific agencies have calculated, under the leadership of WHO (World Health Organization), a daily index of sun irradiance to detect the cut-off value to avoid sunburn and sun risks. Sun light composes of a very wide range of electromagnetic waves of which the human eye is able to perceive light radiations. It knows that MED (minimal erythemal dose) is defined as the quantity of UVB able to elicit an erythemal response. Nevertheless, to calculate skin sensitivity, a new noninvasive method is a multiband spectrophotometer (skin analyzer) with two reflectance measures, and other magnitudes which can be directly correlated to them (e.g., absorbance), a calculation unit and a data storage unit for calculating the corresponding MED. This sensitivity varies from individual to individual and can be determined with different methods [6, 11, 12]. In 2006, Fabbrocini et al. monitored sun radiation to obtain bioclimatic parameters useful for evaluating climatic changes in order to get an adequate sun protection map, set up an experimental photodermatology service, and promote preventive devices through a system of customized UV teledosimetry using information technology such as mobile phones. A total of 450 tourists were selected in Capri town, and they filled a questionnaire containing personal information as sex, age, hair colour, eye colour, phototype, sunscreen used, and sunbathing time. The MED was measured by the Skin Analyser Q Tan, and, after the examination, the team of dermatologists compiled a card for each of them with information about their photosensitivity. The tourists selected in this project before sun exposure were informed by SMSs through their mobile phones or through a light board about the optimal UV dose for their skin and about an estimate of the residual exposure time considering the UV irradiance. More exactly, in the study, the authors correlated the UVI to the phototype of the subjects studied, thus obtaining an estimate of the maximum exposure time expressed in minutes to avoid sunburn. A software instantaneously calculates the exposure time to avoid sunburn on the basis of the effective erythemal dose (DE) expressed in MED/h using the following algorithm ST = 60/DE. Therefore, in a typically sunny August day with a UVI equal to 6 (2.6 MED/h), a phototype I or II at his/her first sun exposure would develop a sunburn after about 20 minutes, while in a moderately pigmented subject this would take some hours [13]. Armstrong et al. also used SMSs as a reminder strategy to improve adherence to sunscreen application. A group of seventy people were enrolled. Half of them received daily SMS reminders through their mobile phones for 6 weeks, and the other half did not receive reminders. The SMSs reminder consisted of two components: a “hook” text detailing daily local weather information and a “prompt” text reminding users to apply sunscreen. This methodology showed that a simple daily reminder could maintain adherence to sunscreen application; in fact the participants who received the SMS reminders were nearly twice as adherent to a regimen of daily sunscreen application respect with control participants who did not receive SMS reminders [14]. Previous studies that had just examined the use of text messaging in health care have found that text-message reminders improve outpatient clinic attendance [15–19], encourage weight loss [20], and provide support to diabetic patients [21]. The results of these studies confirm that text messages or SMSs can promote behavioural changes. In fact, SMSs can be used to promote preventive health behaviours or help individuals adhere to medication regimens for several reasons. The mobile phone renders the people readily available, and the SMS is a simple, low cost, and immediate method of delivering reminders without using the computers, but the point more important is that text messaging may be attractive to younger people, an important target population for development of positive preventive health habits. Despite numerous recommendations, people have yet to understand the problems related to excessive sun exposure and the importance of using sunscreen. The results of these studies suggest that text-message reminders reach a large number of people at low cost. Introduction of a program that incorporates text-message reminders to a large population may be an innovative preventive health measure against the development of skin cancer.

## 3. Diagnosis

Dermoscopy is a noninvasive, in vivo technique and has the potential to improve up to 49% the diagnostic accuracy for melanoma and nonmelanoma skin cancer, if used by experts [22–24]. In 1998, Provost et al. made the first step in the creation of an international teledermoscopy network, using a store-and-forward technology with dermoscopic images [25]. Teledermoscopy represents a recent development of teledermatology. Dermoscopic images of pigmented skin lesions can be transmitted through internet to remote teleconsultants. The feasibility of teledermoscopy reveals a 91% consensus between the face-to-face diagnosis and the remote diagnosis [26]. The Consensus NetMeeting of dermoscopy (a virtual meeting of experts from all over the world) was another evident example of the practical applicability of the use of dermoscopy via the Internet [27]. In 2003, a pilot study of the dermoscopic-pathologic approach using telediagnosis for melanocytic skin neoplasms revealed that the diagnostic accuracy reached 83% versus gold standard (conventional histopathologic diagnosis by experts) [28]. A 2-step teledermatologic approach may be feasible in managing individuals with multiple pigmented skin lesions [29]. Recently, teledermoscopy was evaluated as a filtering system on 219 pigmented skin lesions. Teleconsultations were sent from general practitioners to the pigmented skin lesion clinic of the Department of Dermatology, University of Seville, in Seville, Spain, and 49% of the patients were referred to the FTF Clinic. There was agreement among the teleconsultants for both the diagnosis (*k* = 0.91) and for the management options (*k* = 0.92) [30]. 

Teledermoscopy seems to be suitable as a triage system. In this regard, Tan et al. evaluated if teledermoscopy could be considered as a triage tool of patients referred to a skin lesion clinic. Two-hundred patients referred to a dermatology skin lesion clinic were recruited, and a total of 491 lesions were seen. Digital and dermoscopic photographs were taken of skin lesions of concern, and the patients were then seen independently face-to-face by two out of three dermatologists. The digital images were evaluated 4 weeks later, as a teledermoscopy consultation, by two of these dermatologists. The diagnosis and management from both types of consultation were compared. There was excellent agreement between teledermoscopy and face-to-face diagnosis with only 12.3% of lesions having disparate diagnoses of clinical significance. Only 12 of 491 lesions appeared to have been underreported by teledermoscopy when compared with face-to-face diagnosis. However, when histopathology became available, only one malignant lesion had been missed (a basal cell carcinoma diagnosed as solar keratosis) by teledermoscopy. Teledermoscopy approximated 100% sensitivity and 90% specificity for detecting melanoma and nonmelanoma skin cancers. Importantly, 74% of all lesions were determined to be manageable by the general practitioner without needing to be seen face-to-face by a dermatologist [31].

Massone et al. also investigated the feasibility of teleconsultation using a new generation of cellular phones in pigmented skin lesions. 18 patients were selected consecutively in the Pigmented Skin Lesions Clinic of the Department of Dermatology, Medical University of Graz, Graz (Austria). The face-to-face (FTF) diagnoses (16 benign lesions and 2 melanomas) were made in each case. Clinical and dermoscopic images were acquired using a Sony Ericsson K 750i with a built-in two-megapixel camera and a pocket dermoscopy device with a 25 mm 10x lens *(DermLite II PRO HR (3Gen, LLC—Dana Point, USA))* on which the cellular phone was applied. Two teleconsultants reviewed the images on a specific web application where images had been uploaded in JPEG format. Compared to the face-to-face diagnoses, the two teleconsultants obtained a score of correct telediagnoses of 89% and of 91.5% reporting the clinical and dermoscopic images, respectively [32]. Anyway, in a NHS R&D Technology Assessment, patients referred to a 2-week wait clinic were invited to have a series of digital photographs, with and without dermoscopy, immediately before their face-to-face consultation. A surprisingly high proportion (33%) of referrals proved to have a cutaneous malignancy or a severely dysplastic lesion, with almost 22% having a malignant melanoma or squamous cell carcinoma, possibly reflecting the rise in incidence of skin cancers reported elsewhere. If the highest level of clinician confidence had been applied, no cancers would have been missed, but only 20% of patients would have avoided an outpatient appointment. It was concluded that it is unlikely that this approach can dramatically reduce the need for conventional clinical consultations while still maintaining patient safety [33].

Unfortunately, according to previous observations, teledermoscopy of hypopigmented or nonpigmented lesions cannot always significantly improve diagnostic accuracy. Fabbrocini et al. focused on 44 not very common lesions in the clinical practice, characterized by poor and/or absent pigmentation, absence of regular network, and a diameter <5 mm. The 44 lesions examined by the two observers consisted of 39 melanocytic lesions (12 Spitz naevi, 9 melanomas, 8 melanomas *in situ, *7 Clark naevi, 2 dermal naevi, and 1 Spitzoid melanoma) and 5 nonmelanocytic lesions (2 seborrheic keratosis, 1 dermatofibroma, 1 haeman-gioma, and 1 Bowen papulosis). All the lesions were histo-pathologically confirmed after clinical and dermoscopic diagnoses. They chose these kinds of lesions with high diagnosis complexity either clinically or dermoscopically to evaluate the reliability of using teledermoscopy for the diagnosis of rare and atypical lesions. The pattern analysis and the 7-point checklist were applied to all 44 skin lesions for the early detection of melanoma. The interobserver agreement and the agreement between face-to-face diagnosis and telediagnosis were assessed. The agreement was investigated using Cohen's *K* statistics. With respect to the pattern analysis, it was observed that some features, such as leaf-like areas, milia-like cysts/comedo-like openings, blue-white structures, and blotches, are detected with the same frequency in face-to-face and teledermoscopic observations. Regarding pigment network, regression structures, and diffuse pigmentation, the results showed that they were more evident in teledermoscopic observation, whereas vascular pattern, radial streaks, and dots/globules had lower detection frequency. With respect to the 7-point checklist, it was noticed that irregular pigmentation and regression structures had a higher frequency in teledermoscopic observation; all other criteria were better detected in face-to-face observation. A significant difference in total score derived from 7-point checklist analysis was evident between face-to-face observation and teledermoscopic observation; lesions with a score equal or superior to 3 were found mostly in face-to-face observation. The agreement, evaluated through Cohen's *K*, between the 1st and the 2nd observer on clinical diagnosis was very low (*K* = 0.362). Moderate agreement (*K* = 0.435) was detected between the 1st and the 2nd observer on dermoscopic diagnosis; this suggested that dermoscopy improved clinical diagnosis. this suggested that dermoscopy improved clinical diagnosis. These data showed that telediagnosis, compared with face-to-face diagnosis, presented a lower diagnostic accuracy [34].

## 4. Conclusion

Teledermatology has gained a lot of interest among scientific community. It can deeply revolutionize the delivery of dermatology services, providing equitable services to remote areas and allowing primary care physicians to refer patients to well-equipped dermatological services at a distance. Images can be transmitted via a virtual private network to the teledermoscopists, and those ones can be made mobile as the technology is portable, overcoming geographical barriers, and delivering service to remote areas [35–37].

In particular, teledermatology can aid in the prevention and diagnosis of nonmelanoma and melanoma skin cancer. Patients seem to accept teledermatology, considering it as an excellent mean to obtain healthcare, particularly in those areas with no expert dermatologists available. The use of innovative mobile technology can improve prevention campaign, reaching young people too [38]. Clinicians have also generally reported affirmative experiences with teledermatology, excluding for the diagnosis of “pink” lesion. Further studies focusing on cost effectiveness, intrinsic limits, and patient and clinician satisfaction will facilitate to delineate the potential of teledermatology as a mean of dermatologic healthcare delivery.
